# An Update on the Effects and Complications of BoNT-A in the Management of Third, Fourth, and Sixth Nerve Palsies: A Narrative Review

**DOI:** 10.18502/jovr.v20.14666

**Published:** 2025-06-24

**Authors:** Mohammad Reza Talebnejad, Roghayyeh Baghban, Alireza Attar, Aidin Meshksar, Mansoureh Bagheri, Mohammad Reza Khalili

**Affiliations:** ^1^Poostchi Ophthalmology Research Center, Department of Ophthalmology, School of Medicine, Shiraz University of Medical Sciences, Shiraz, Iran; ^2^Health Sciences North Research Institute, Northern Ontario School of Medicine University, Ontario, Canada

**Keywords:** Botulinum Toxin Type A, Cranial Nerve, Motor Nerve Palsy

## Abstract

This review article explores the etiology of oculomotor palsies—including third, fourth, and sixth cranial nerve palsies—and addresses the application of botulinum toxin type A (BoNT-A) in the management of these conditions, along with its associated complications and side effects. The objective is to assess BoNT-A's potential efficacy and its role across various types of nerve palsies. A comprehensive analysis of relevant studies reveals that BoNT-A holds promise as a therapeutic option in managing these conditions. BoNT-A injection into the lateral rectus muscle proves to be an effective treatment for addressing post-traumatic third nerve palsy. This is achieved by providing symptom relief and diminishing the necessity for subsequent surgical interventions. In the context of fourth nerve palsy, BoNT-A injection into the inferior oblique or inferior rectus muscles presents potential benefits but is accompanied by certain limitations.

Additionally, previous studies have shown that BoNT-A injection into the antagonist medial rectus muscle for treatment of sixth nerve palsy results in favorable outcomes, such as contributing to functional improvement. The literature highlights the importance of timing, dosage, and grade of muscle dysfunction when administering BoNT-A injections. BoNT-A injection is an effective option to manage different types of cranial nerve palsies and improve binocular function. Furthermore, it plays an integral role in preventing antagonist muscle contracture and, hence, the need for future surgical intervention.

##  INTRODUCTION

Oculomotor nerve palsy frequently leads to diplopia and commonly results in a referral to a neuro-ophthalmology clinic, particularly when associated with visual loss. Indeed, the prognosis for these conditions is usually favorable, but timely identification of the underlying cause is crucial for effective management.^[1,2]^ Various kinds of cranial nerve palsy (CNP) present with a wide range of manifestations. For example, third nerve palsy might result in pupillary involvement, ptosis, aberrant regeneration, amblyopia, incomplete Bell's phenomenon, lateral rectus (LR) contracture, and superior oblique (SO) overaction.^[3]^ Fourth nerve palsy frequently causes vertical diplopia due to ipsilateral hypertropia that increases in contralateral gaze or head tilt toward the affected side.^[4]^ The prevalence of third, fourth, and sixth nerve palsies varies depending on underlying causes and population demographics. Third nerve palsy is relatively uncommon compared to other oculomotor nerve palsies, while sixth nerve palsy is the most common.^[5]^ In adults, third, fourth, and sixth cranial nerve palsies often arise from suspected ischemia affecting the nerve, particularly in the presence of microvascular risk factors such as diabetes mellitus, older age, hyperlipidemia, and hypertension. With the advent of magnetic resonance imaging, less benign yet treatable causes have been identified, including aneurysms, intracranial tumors, infections, inflammation, and brainstem infarctions.^[6]^

BoNT-A shows promise in addressing challenges linked to third, fourth, and sixth nerve palsies, which are characterized by impaired eye movement coordination. This nonsurgical approach provides controlled muscle weakening, thereby enhancing ocular alignment. Noted for its therapeutic effectiveness, BoNT-A offers a reversible approach to managing diverse ocular nerve palsies, demonstrating its utility in addressing the unique challenges of these conditions.^[7,8,9]^ BoNT-A can also be used as an adjunct to transposition surgery or as temporary relief for the patient's diplopia.^[10]^ This article presents a thorough review and assessment of the role of BoNT-A in managing third, fourth, and sixth nerve palsies.

### Botulinum Toxin Type A: Mode of Action and Clinical Applications

BoNT-A is a potent toxin produced by the anaerobic bacterium called *Clostridium botulinum*.^[11]^ BoNT-A gained FDA approval in 1989 for treating strabismus, blepharospasm, and hemifacial spasm.^[12]^ The mechanism of action of BoNT-A involves the inhibition of presynaptic acetylcholine release by binding to specific receptors on the cell surface and, ultimately, disrupting the normal signaling between motor neurons and muscle fibers.^[13,14,15,16]^ The effect of BoNT-A is reversible, allowing for adjustments in dosage and placement if future injections are required to achieve optimal results. Selectively weakening a specific eye muscle helps restore proper eye alignment and alleviate associated symptoms such as diplopia. The nonsurgical, reversible nature of BoNT-A injections makes them an attractive therapeutic option that can improve patients' quality of life.^[7,8,9][17][18]^

##  ETIOLOGY 

### Oculomotor Nerve Palsy 

Cranial nerve III palsy (CNP III) often presents with ptosis, mydriasis, exotropia, and hypotropia of the affected eye. Acquired third nerve palsy has a diverse array of etiologies, and understanding these factors requires differentiation between pediatric and adult populations. In adults, various conditions such as microvascular damage, inflammation, autoimmune disorders, viral or bacterial infections, tumors, aneurysms, iatrogenic damage from neurosurgical procedures, and head trauma are commonly implicated in isolated third nerve palsy.^[19]^ Microvascular ischemia has been specifically highlighted by Canady et al as a prevalent factor in cases without pupil involvement.^[20]^ In contrast, Miller et al emphasized that in pediatric cases, the primary cause of third nerve palsy is often congenital. Acquired instances in children are less frequent, with trauma and infection being the leading contributors, followed by neoplasms, aneurysms, and ophthalmoplegic migraine. This division into pediatric and adult categories underscores the importance of age-specific considerations in assessing and managing third nerve palsy. The etiological spectrum in adults encompasses a broader range of vascular and acquired conditions, while congenital factors predominate in children.^[21]^

### Trochlear Nerve Palsy 

Isolated acquired cranial nerve IV palsy (CNP IV) is less common than oculomotor and abducens nerve palsies,^[22]^ but congenital fourth nerve palsy is very common.^[23]^ Acute SO muscle palsy leads to hypertropia and ocular torsion, possibly resulting in symptoms such as diplopia and an unusual head posture. Among etiological factors, trauma stands out as the most recognized one, yet idiopathic etiology remains the predominant cause overall. Other underlying conditions leading to CNP IV include viral and bacterial infections, inflammatory conditions due to autoimmune diseases, diabetes mellitus, and—to a significantly lesser extent—intracranial space-occupying lesions, also noted in CNP VI cases.^[24]^

### Abducens Nerve Palsy 

Inflammation is the most common cause of CNP VI in adults. Other important causes include space-occupying lesions such as pituitary macroadenoma, carotid artery aneurysm, sphenoidal sinusitis, and sinonasal carcinoma that extends into the cavernous sinus.^[24]^ Ischemia linked to risk factors such as diabetes mellitus and hypertension is also identified as a cause of CNP VI in adults. Tumors are the most common cause of CNP VI in the pediatric population, whereas trauma and vascular causes are more frequently observed in adults.^[25,26,27]^ Furthermore, congenital CNP VI is underreported in pediatric cases and is often misdiagnosed as infantile esotropia, as it typically resolves within the first four to six months of birth.^[28]^ Investigating the causes of sixth nerve dysfunction in children under the age of seven, Aroichane et al reported hydrocephalus as the most common cause, followed by trauma, congenital conditions, and viral infections.^[29]^

### Surgical Technique

BoNT-A can be injected under local anesthesia, although general anesthesia is preferred in certain patients, especially children. However, most general anesthetic drugs can interfere with EMG signals due to their muscle relaxant effects. Topical tetracaine eye drops are used for local anesthesia, followed by the application of a sponge soaked with lidocaine placed over the injection site. For maximal paralytic effect, it is preferable to inject the toxin near the neuromuscular junction, where the nerve innervates the muscle. The most common method is EMG-guided injection, especially when treating inferior rectus (IR) or inferior oblique (IO) muscles due to their special anatomy and limited accessibility. Once the EMG electrodes are positioned on the patient's forehead and local anesthesia is administered, the patient is asked to look in the opposite direction of the action of the muscle intended for injection. After grasping the muscle with forceps, a 27- or 30-gauge needle is introduced into the muscle transconjunctivally, superficial to sclera and 5 mm away from the muscle's insertion site or, in case of sixth nerve palsy, 1 cm away from the limbus. Then, the patient is asked to look in the direction of action of the muscle, which would result in an increased EMG output; thereafter, the toxin is slowly injected.^[30,31,32]^ The treatment dosage is determined by the extent of deviation and the restriction in muscle movement. To reduce the risk of drug leakage and dispersion, the needle is then gradually withdrawn after 10 to 15 seconds.^[33]^ Despite EMG guidance, BoNT-A injection is especially challenging in eyes with previous retinal detachment surgery or high myopia.^[34]^

There are other less common ways to inject the toxin, such as injection without EMG guidance, which has been reported to be safe and as effective as the EMG-guided approach,^[32,35,36]^ or injection under direct visualization with conjunctival incision.^[30,37]^ Also, subtenon injection of BoNT in patients with acute sixth nerve palsy has been shown to be as effective as EMG-guided intramuscular injection, with the advantage of avoiding the difficulties of EMG. Subtenon injections were applied around 10 mm away from the nasal limbus with a 27-gauge needle, and there was no reported adverse effect except for mild subconjunctival hemorrhage.^[38]^ An animal study showed that subtenon injection of BoNT in acute sixth nerve palsy resulted in morphological changes of extraocular muscles, comparable to intramuscular injection.^[39]^ Therefore, subtenon injection of BoNT could be a safe and quick alternative for intramuscular injection in patients with acute sixth nerve palsy and could reduce the recovery time in these patients.^[40]^

### Botulinum Toxin Type-A Treatment of Third Nerve Palsy 

In our previous study, the effects of BoNT-A (Dysport, Ipsen Biopharm Ltd, Wrexham, UK) on paralytic exotropia secondary to traumatic CNP III were evaluated. The study involved nine patients who had third nerve palsy with a duration of less than two months. In this study, LR injections were administered, and each patient received 20 units of Dysport. The maximal effect occurred at two weeks post-injection. The recovery rate significantly improved to < 10 prism diopters (PD), and single binocular vision was restored in the primary position, and the mean angle reduced from 48.3 to 14.2 PD. BoNT provided swift short-term relief for traumatic third nerve palsy.^[41]^ Saad et al examined 10 patients with isolated partially recovered CNP III. In their case series, head trauma was a common cause of third nerve palsy, impairing the binocular fusion. In their study, 75% of patients with third nerve palsy experienced improved binocular single vision after the administration of BoNT-A injection into the LR muscle. The authors concluded that in cases with established fusion and adequate adduction of the affected eye, injecting BoNT into the LR muscle may provide long-lasting control over ocular deviation. However, their study suggested that BoNT-A toxin injection into the LR does not consistently predict MR function or the success of squint surgery [Table 1].^[42]^

**Table 1:** Studies conducted on the effects of BoNT-A in third nerve palsy

**Study**	**Type of study**	**Method**	**Population sample size**	**Outcome measures**	**Findings**
Talebnejad et al, 2008^[41]^	Case series	Patients with third nerve palsy ( < 2-month duration) received BoNT injections; Recovery criteria: < 10 PD exotropia or no diplopia	9 patients who had third nerve palsy after head trauma	Pre-injection: 48.3 PD exotropia; Post-injection: 14.2 PD; 77.8% recovered; two required surgery	BoNT-A injection into the lateral rectus muscle substantially improves recovery in acute third nerve palsy within two months
Saad et al, 1992^[42]^	Case series	Two groups: Therapeutic (stable alignment) and Diagnostic (predicting surgery outcome)	10 patients with a partially recovered third nerve palsy	BoNT had diverse effects on patients with third nerve palsy, particularly influencing their ocular alignment and fusion potential	BoNT aids in adduction and fusion recovery in patients with exotropia due to third nerve palsy

### Treatment of Fourth Nerve Palsy using Botulinum Toxin Type-A

BoNT-A is cautiously utilized for CNP IV, as surgical outcomes can be unpredictable, carrying a risk of over- or under-correction. In a study on 20 patients with unilateral or bilateral fourth nerve palsy, Garnham et al evaluated two groups. Group 1 consisted of 10 patients who received primary BoNT-A therapy. Six patients who had BoNT-A injection into the IO muscle experienced limited benefits, requiring surgery (83%) or prism use (17%) eventually. However, five patients who received BoNT-A injection into the IR muscle became symptom-free. Of the 10 patients in group 2 who received BoNT-A postoperatively, all but 1 without fusional ability experienced long-term benefits. This comparative study showed that primary BoNT-A use for chronic CNP IV may be of limited value; however, it could be of greatest benefit when injection is performed into the IR muscle in patients with residual deviation.^[43]^ In our previous study, we conducted a prospective case series to evaluate the results of BoNT-A injection into the ipsilateral IO muscle for early management of acute traumatic SO muscle palsy. All participants enrolled in the study exhibited IO muscle overaction. Thirteen patients received 10–20 units of Dysport within four weeks of the incident. The study revealed that a single BoNT-A injection significantly reduced hypertropia, IO overaction, and torsion, with 77% of patients becoming diplopia-free within a month. Those with less initial hypertropia had better outcomes. This suggests that BoNT-A is a safe and rapid solution, particularly for patients with baseline hypertropia of 10 PD or less [Table 2].^[44]^

**Table 2:** Studies conducted on the effects of BoNT-A in fourth-nerve palsy

**Study**	**Type of study**	**Method**	**Population sample size**	**Outcome measures**	**Findings**
Talebnejad et al, 2015^[44]^	Prospective case series	Traumatic SO palsy patients received 10–20 BoNT-A units within four weeks	13 consecutive patients with unilateral acute traumatic SO palsy	Hypertropia decreased significantly after BoNT-A injection, with improved IO overaction and subjective torsion, resulting in 77% of patients becoming diplopia-free	A single BoNT-A injection into the IO muscle can promptly and safely alleviate symptomatic diplopia in acute traumatic SO palsy, facilitating recovery
Garnham et al, 1997^[43]^	Comparative study	Patients with CNP IV received BTXA injections in inferior oblique or rectus muscles; Fifty percent had prior surgery, and follow-up averaged 19 months	20 patients, aged 19–70 years	Group I: BoNT-A primary therapy, limited success with oblique injection; Group 2: BoNT-A for post-op deviations, mainly successful with rectus injection	BoNT-A most effective for residual deviations, especially with inferior rectus injection, but less useful as primary therapy in chronic CNP IV N
BoNT-A, BoNT A; CNP IV, fourth nerve palsy; IO, inferior oblique; SO, superior oblique					

### Treatment of Sixth Nerve Palsy using Botulinum Toxin Type A

Metz et al investigated the effectiveness of BoNT-A injection in managing both unilateral and bilateral CNP VI. In their research, including 23 patients with acute unilateral and 11 patients with bilateral CNP VI palsy primarily due to traumatic causes, injections were administered into the MR muscle within three months of the incident. Patients over 13 years of age (mean age: 55 years) received 2.5 to 10 units of BoNT-A, adjusted for deviation size, and were followed up for an average of 11 months. Notably, among 45 patients with unilateral CNP VI who did not receive botulinum therapy within three months, 30% experienced spontaneous recovery. In contrast, in the group of 31 patients who received botulinum treatment, 90% of those with unilateral palsy achieved fusion, while 64% of those with bilateral palsy required surgical intervention, suggesting a less favorable prognosis.^[18]^

In a similar study, Fitzsimons et al categorized patients into two groups. In Group A, the patients were treated solely with BoNT-A injections, resulting in a functional cure in 37% of cases, although one patient with bilateral palsy required three injections. In Group B, over 50% of patients had traumatic CNP VI, 80% of which were bilateral. These findings suggest that BoNT can play a significant role in managing sixth nerve palsy and may even have a preventive impact against contracture.^[45]^

The effectiveness of BoNT-A injections for sixth nerve palsy is influenced by the chronicity of the condition, as highlighted in studies by Holmes et al^[46]^ and Murray et al.^[47]^ Holmes et al focused on patients who had chronic sixth nerve palsy for over six months. Success, defined as the absence of diplopia at distance fixation, was achieved in 12% of patients and partial success ( ≤ 10 PD misalignment) in another 12%, while 75% experienced failure (percentages are rounded to the nearest whole number). Although BoNT-A demonstrated some efficacy in chronic cases, the study emphasized that surgery is often necessary to achieve long-term binocular vision.^[46]^ According to Murray et al, early BoNT-A treatment (within eight weeks) led to rapid fusion and complete recovery in six out of ten patients, while delayed treatment resulted in incomplete recovery, with persistent lateral rectus weakness in one case. Early intervention with BoNT-A appeared beneficial, particularly for rapid recovery, but chronic cases with poor lateral rectus function often required surgical intervention.^[47]^ The prospective study by Holmes et al on chronic CNP VI compared various approaches, revealing success rates of 15% with conservative treatment, 10% with BoNT, 39% with surgery, and 25% with a combined approach. Notably, botulinum treatment alone was rarely successful, reinforcing the often essential role of surgical intervention in managing chronic CNP VI.^[48]^

Merino et al conducted a 14-year retrospective study on pediatric patients with CNP VI. Neoplasms were the leading cause, and BoNT successfully treated seven of ten patients. Three patients required surgery, and recovery took 39 months. While spontaneous recovery occurred in one-third of patients, most required BoNT treatment, and surgery was successful with a single procedure.^[27]^

Kerr et al investigated the effectiveness of BoNT-A injections in children with CNP VI caused by brain tumors. Of the 19 children, 10 underwent conservative management, and 2 of them experienced recovery without surgical intervention. In the group of nine children who received BoNT-A, two (22%) recovered without surgery. The initial response to BoNT-A showed low recovery rates, similar to the untreated group. Brain tumor-related palsies had limited recovery, possibly due to persistent lateral rectus muscle dysfunction. BoNT-A did not provide a permanent reduction in esotropia but did improve binocular function in treated children.^[49]^

Considering the adult population, Ganesh et al evaluated the use of BoNT-A for early treatment of CNP VI in patients with type 2 diabetes. Their study involving 31 cases showed that BoNT-A injections significantly improved head turn, ocular deviation, and abduction. A high success rate of 83.9% was observed, and 90.3% of patients achieved symptom resolution. This early BoNT-A injection effectively accelerated recovery by preventing MR contracture. According to the authors, BoNT-A is a safe and efficient treatment option for diabetic patients with CNP VI, ultimately improving their quality of life and reducing the need for future surgical interventions.^[50]^ In another study, eight patients with intracranial malignancies or vascular lesions and CNP VI received BoNT treatment for chemodenervation of the antagonist MR muscle. Primary deviation ranged from 20 to 75 PD of esotropia. Acute treatment showed success, resulting in near full abduction recovery; besides, most cases achieved diplopia-free condition associated with excellent rotations during the 20.6-month follow-up period.^[51]^ Such findings indicate the potential use of BoNT in addressing specific etiologies associated with intracranial lesions.

Moreover, Díaz-Maroto studied the outcomes of BoNT treatment in patients with CNP VI caused by trauma or tumors. Group I (trauma) had a 38% success rate within six months and exhibited a lower initial deviation, and Group II (tumors) achieved a 57% success rate. The authors observed that timely BoNT treatment is beneficial for traumatic palsies, although initial deviation and muscle function can impact the outcomes. It is also a valuable chronic treatment and diagnostic procedure.^[52]^ Sugano et al treated esotropia in 20 patients using BoNT-A (Prosigne, Lanzhou Institute of Biological Products, China). The procedure was performed on both medial rectus muscles in 15 pediatric patients with esotropia of < 50 PD. Furthermore, it was administered to five adults with esotropia secondary to sixth nerve paralysis or paresis, with a focus on targeting a single MR muscle. Success rates declined from 61% to 50% between the third and sixth months of follow-up. Furthermore, BoNT-A showed no improvement in acute CNP VI, possibly due to inadequate dosage and delayed care.^[53]^

We previously investigated the use of BoNT-A as an alternative to surgery for acute complete CNP VI. BoNT-A injections (1–10 units Dysport) were administered into the MR muscle within one month of onset in 30 patients aged 9 months to 70 years. Follow-up assessments on days 1, 7, 30, 90, and 180 measured abduction capability, binocular field of vision, and the extent of residual deviation. The results showed that 73% of patients (22 individuals) achieved a diplopia-free binocular field exceeding 75º and had residual esotropia of < 10 PD, while the remaining 27% (8 patients) had residual esotropia that ranged from 10 to 50 PD, requiring surgery. Among treatment failures, defined as those with esotropia of > 10 PD, CNP VI was attributed to tumors in two patients and trauma in the other two. Notably, there were no cases of globe perforation or exotropia.^[54]^ In another study, 48 eyes with sixth nerve palsy—attributed to ischemia, trauma, and inflammation—received BoNT injections. The results showed that 83.9% of fully resolved cases achieved binocular function, confirming BoNT's efficacy.^[55]^ Murray et al investigated the effects of BoNT injection on eight patients with complete CNP VI. Of these, seven cases resulted from head trauma, while one was due to cerebrovascular disease. Within eight weeks of palsy onset, seven patients achieved rapid fusion and full function recovery, showing no diplopia or confusion. Early intervention seemed beneficial, but natural recovery and diverse etiologies warrant a double-blind study for accurate therapy assessment.^[56]^ Metz et al studied 29 cases of acute unilateral sixth nerve palsy treated with BoNT injection, which was administered into the antagonist MR. Most patients achieved complete motility recovery (76%). Among the seven patients exhibiting a residual abduction deficit, two demonstrated fusion in the primary position, three achieved fusion with prismatic correction, and two required subsequent surgical intervention.^[57]^ In conclusion, understanding the diverse outcomes of BoNT injections for CNP VI requires considering age and the specific underlying etiology of the palsy.

Studies comparing conservative management versus BoNT injection for CNP VI have reported variable results. Holmes et al conducted a multicenter study on 84 patients with acute traumatic CNP VI. They found similar recovery rates between the two treatment groups (71% with conservative treatment and 73% with BoNT injection), indicating comparable outcomes of BoNT injection versus conservative care in managing acute CNP VI.^[58]^ In contrast, Hung et al examined the use of BoNT-A injection for acute unilateral complete CNP VI caused by head trauma. BoNT-A-treated patients (*n* = 14) had a significantly higher recovery rate (64.3%) compared to the conservative treatment group (26.3%, *P* = 0.028). The authors concluded that BoNT-A improves recovery in severely injured patients with acute traumatic CNP VI.^[59]^ Furthermore, a randomized trial assessed the effect of early BoNT-A injection on recovery in acute unilateral CNP VI. Among 47 patients, 22 received injections and 25 were in the control group. Both groups were recruited for the study within three weeks following the initial presentation of the symptoms. Recovery rates were 80% in the control group and 86% in the treatment group, suggesting no prophylactic effect of BoNT in the studied population.^[60]^

**Figure 1:**
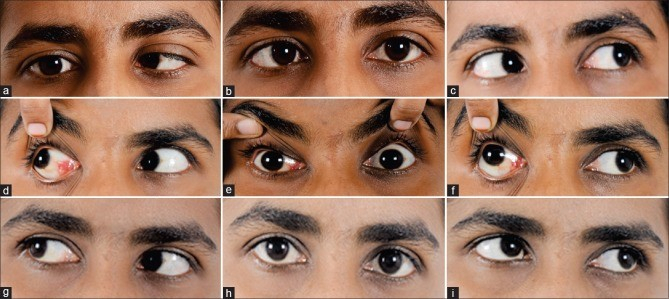
A 32-year-old woman presented with right lateral rectus palsy and –4 abduction limitation (a–c). The patient experienced ptosis, exotropia, and adduction limitation 4 days following BoNT injection into the right medial rectus muscle (d–f). She achieved complete resolution of all symptoms one month after BoNT injection (g–i).^[63]^.

Numerous methods have been explored for administering Botox in treating CNP VI. Kao et al studied the effectiveness of subtenon BoNT injection for acute traumatic CNP VI. In 13 patients with < 6 months of palsy, post-injection deviation was reduced from 39.5 to 17.0 PD. Recovery ( < 10 PD deviation or absence of diplopia) was seen in 53.8% of the patients (unilateral 63.6%, bilateral 0%). According to this study, subtenon BoNT treatment offers superior recovery rates for traumatic CNP VI, particularly in unilateral cases.^[61]^ Chen et al studied the effect of BoNT-A injections for acute esotropia attributed to CNP VI. Fifteen patients with early onset CNP VI received subtenon injection of BoNT-A into the MR without EMG guidance. The results showed 40% full recovery, 13% partial recovery with prism, and 47% requiring surgical intervention. The authors reported minimal complications, reinforcing the safety and effectiveness of BoNT-A [Figure 1; Table 3].^[62]^

**Table 3:** Studies on the effects of BoNT-A in sixth nerve palsy

**Study**	**Type of study**	**Method**	**Population sample size**	**Outcome measures**	**Findings**
Talebnejad et al, 2003^[54]^	Case series	Patients aged 9 months to 70 years received BoNT-A injection	30 patients	80% improved abduction; 73% achieved diplopia-free field; 27% needed surgery	BoNT-A improved abduction in sixth nerve palsy
Kerr et al, 2001^[64]^	Comparative study	Explored BoNT's impact on sixth nerve palsy outcomes in children with brain tumors (1992–1999)	19 children	10 were conservatively managed; 2 (20%) recovered; 9 received BoNT; and 2 (22%) recovered without surgery	BoNT did not enhance recovery in children with sixth nerve palsy due to brain tumors
Fitzsimon et al, 1989^[45]^	Case series	Patients with sixth nerve palsy using BoNT, either alone or with muscle surgery; Assessing benefits and complications	55 patients	BoNT benefited 72% of patients with sixth nerve palsy	BoNT treatment proved significantly beneficial
Ganesh et al, 2019^[63]^	Case series	Patient with type 2 diabetes and acute onset of sixth cranial nerve palsy, who received BoNT injection into medial rectus muscle	31 cases	BoNT significantly improved sixth nerve palsy, with 90.3% achieving full resolution and 83.9% successful treatment	Early BoNT-A is safe, effective, avoids surgery, and improves life quality in diabetic patients with sixth nerve palsy
Holmes et al, 2000^[58]^	Clinical trial	Traumatic sixth nerve palsy, BoNT group received injection within three months; Recovery criteria at six months was assessed, excluding cases with less than six months of follow-up	84 patients	74% conservative treatment, 26% BoNT, similar recovery rates	BoNT and conservative treatments had similar high recovery rates in patients
Holmes et al, 2021^[46]^	Case series	Patients with chronic sixth nerve palsy received initial BoNT treatment; Success was defined as no diplopia and partial success within 10 PD	10 patients	12% success, 12% partial success, 75% failure	BoNT is somewhat effective but often requires surgery for chronic sixth nerve palsy
Hung et al, 2005^[59]^	Retrospective study	Comparing the effect of BoNT versus conservative treatment on acute sixth nerve palsy	33 patients	BoNT group had higher recovery rate (64.3% versus 26.3%)	BoNT aids recovery in severe acute traumatic complete sixth nerve palsy
Kao et al, 2003^[61]^	Comparative study	Patients with traumatic sixth nerve palsy treated with BoNT; recovery was defined as < 10 PD esotropia or no diplopia at three months	13 patients	7 (53.8%) recovered; 6 needed surgery	BoNT for traumatic sixth nerve palsy yielded better recovery rates than conservative treatments; unilateral cases had superior outcomes
Lee et al, 1994^[65]^	Clinical trial	The effect of ipsilateral medial rectus treatment on unilateral sixth nerve palsy: 22 patients received injections; 25 were controls	22 patients	83% of patients began treatment within two weeks, with recovery rates of 80% in the control group and 86% in the injection group.	BoNT lacked a preventive effect; Recovery rates remained similar between groups
Mets et al, 1991^[66]^	Clinical trial	Average onset-to-treatment interval: 40 days; mean follow-up post-injection: 14 months	29 patients	76% achieved complete recovery; 7 with residual deficits had various outcomes	BoNT is effective for acute unilateral sixth-nerve palsy treatment
Murray et al, 1989^[47]^	Case series	BoNT treatment for sixth nerve palsy, followed for 14 months	10 patients	Early BoNT improved fusion in acute sixth nerve palsy; long-term benefit uncertain	BoNT was ineffective for cases with poor lateral rectus function
Murray et al, 1991^[67]^	Case series	BoNT for total sixth nerve palsy, within eight weeks of onset	8 patients	7 of 8 gained fusion, recovered fully with BoNT	Early BoNT helps sixth nerve palsy
Wagner et al, 1989^[68]^	Comparative study	Patients with intracranial lesions and sixth nerve palsies treated with BoNT for esotropia	8 patients	6 treated acutely, 2 after partial recovery; most achieved excellent outcomes	BoNT improved sixth nerve palsies
Chuenkongkaew et al, 2001^[69]^	Case series	BoNT treated 48 sixth nerve palsy eyes due to ischemia, trauma, and inflammation	45 patients	Group I ( ≤ 24 weeks) had better recovery, 71.1% complete resolution, binocular function in 83.9%	BoNT is a safe, effective alternative treatment for acute sixth nerve palsy.
Liano sanches et al, 2000^[70]^	Case series	21 patients in group I had traumatic sixth nerve palsy, while 14 in group II had tumoral etiology. Both groups received BoNT treatment, with parameters analyzed	35 patients	In group I, mean number injections: 1.7, dose: 10.23 IU, 38% success; Group II: mean number injections 1.5, dose 8.21 IU, 57% success, no significant influence among factors observed	BoNT was effective for traumatic palsy within six months, dependent on deviation and muscle function; also useful for chronic treatment and diagnosis
Merino et al, 2010^[71]^	Retrospective study	A 14-year study on children with sixth nerve palsy: treatment with BoNT, followed by surgery if needed, led to success with orthotropia and no diplopia	15 patients	BoNT effectively managed pediatric sixth nerve palsy, with successful outcomes in most cases and some requiring follow-up surgery	Neoplasms emerged as the predominant cause of sixth nerve palsy in the cohort; Spontaneous recovery occurred in one-third of cases, while the majority required BoNT treatment, often yielding successful outcomes; Surgical intervention, when required, was successful with a single procedure

An overview of the reported studies reveals that sixth cranial nerve palsy (CNP VI) is relatively common compared to third and fourth nerve palsies, because the sixth cranial nerve is more prone to damage owing to its proximity to the skull base and its small size.^[72]^ Moreover, the sixth cranial nerve has a long and tortuous course, which makes it susceptible to lesions at various points along its path.^[73]^ Both fourth and sixth nerve palsies typically involve a single extraocular muscle—the SO and the lateral rectus, respectively. However, botulinum toxin injections are more commonly used in sixth nerve palsy than in fourth nerve palsy. This difference may be attributed to the greater likelihood of significant esotropia and diplopia in sixth nerve palsy, which often necessitates more active intervention. The selective action of BoNT-A on specific muscles makes it an effective option for weakening the overactive muscles and restoring proper eye alignment. In CNP VI, the LR muscle is predominantly affected, and BoNT-A can be used to weaken the MR muscle, helping to alleviate eye misalignment and double vision.^[75]^ On the other hand, CNP III involves multiple eye muscles with complex interactions. In cases of third nerve palsy, various muscles responsible for different eye movements, including elevation and adduction, can be affected.^[76]^ Moreover, BoNT-A effects are temporary, typically lasting several months. This is advantageous for conditions like CNP VI, where the underlying nerve dysfunction may eventually improve on its own over time.^[72,77,78,79]^ The reversible effects of BoNT-A allow for adjustments in future management depending on the patient's condition and response to treatment.^[80,81]^

### Complications 

Sugano et al reported transient complications such as ptosis (38.89%) and vertical strabismus (11.11%) after injection of BoNT-A into the MR muscle.^[82]^ Talebnejad et al also reported transient lower lid ptosis, transient hypertropia, and subconjunctival hemorrhage after BoNT-A injection into the IO muscle. Notably, major complications such as globe perforation were not observed in either study.^[44]^ However, Levy et al discussed the rare occurrence of persistent mydriasis after BoNT-A injections, which can result from direct trauma to the ciliary ganglion, intraconal BoNT-A diffusion, and damage to pupillary sphincter muscles.^[83]^ These studies emphasize the importance of meticulous surgical skills, accurate dosage determination, and careful monitoring after BoNT-A injection. The latter involves postoperative follow-up visits and assessments to ensure the safety and effectiveness of the treatment. It also includes evaluating the persistence of paralysis and muscle relaxation effect, as well as assessing the depth and recovery of neuromuscular blockade during anesthesia. Regarding dosage determination, experts suggest administering 2.5 to 5 units of BoNT-A to achieve sufficient IO paresis while minimizing complications.^[84,85]^

##  CONCLUSIONS, CHALLENGES, AND PERSPECTIVES

The application of BoNT-A for treating third, fourth, and sixth nerve palsies represents a dynamic and evolving field that continues to show promising results. BoNT-A's mechanism of action— involving the targeted inhibition of neurotransmitter release at neuromuscular junctions—presents a precise and controlled approach to muscle weakening. BoNT-A holds significant potential in effectively addressing the challenges posed by third, fourth, and sixth nerve palsies, conditions characterized by impaired eye movement coordination. By providing controlled muscle weakening and facilitating improved ocular alignment, BoNT-A could be used as a minimally invasive procedure that reduces the need for traditional surgical interventions. Moreover, the effects of BoNT-A are temporary, typically lasting several months. The implementation of BoNT-A for the treatment of ocular nerve palsies necessitates a judicious approach. Careful administration, proper dosing, and continuous monitoring are essential to achieve optimal outcomes and ensure patients' safety. Long-term studies are imperative to comprehensively evaluate the sustainability of BoNT-A's effects and refine dosing protocols.

##  SUMMARY

This review highlights BoNT-A's potential as a promising therapy for third, fourth, and sixth cranial nerve palsies. BoNT-A improves binocular function, prevents muscle contracture, and reduces the need for surgery. However, its efficacy depends on timing, dosage, and muscle dysfunction severity, with varied outcomes depending on the type of nerve palsy under treatment.

##  Ethical Considerations

This study was approved by the Ethics Committee of Shiraz University of Medical Sciences (Ethics code: IR.SUMS.REC.1403.070).

##  Financial Support and Sponsorship

None.

##  Conflicts of Interest

None.
